# The Retrochiasmal Optic Pathway: A Link in Jeopardy

**DOI:** 10.3389/fsurg.2020.00035

**Published:** 2020-06-23

**Authors:** Manuel Campos, Allan J. Drapkin

**Affiliations:** ^1^Department of Neurosurgery, Clínica Las Condes, Santiago, Chile; ^2^Department of Surgery [Neurosurgery], Jersey Shore University Medical Center, Neptune, NJ, United States

**Keywords:** homonymous hemianopia, meningioma, visual hallucinations, optic radiations, retrochiasmal optic pathway

## Abstract

The case of an intraventricular meningioma is presented and the visual complication incurred by its surgical resection is discussed. The importance of selecting the most optimal surgical approach and the basis for that selection are highlighted.

## Background

Intraventricular tumors are uncommon and account for only 1.4% of all primary central nervous system tumors (1). Approximately, half of these occur in the lateral ventricle (2) and are often slow growing and benign. Clinically they frequently present with non-specific symptoms such as headache, cognitive impairment and/or gait disturbances that eventually lead to imaging studies that establish their diagnosis. Depending on its location within the ventricular system, different surgical approaches have been suggested in an attempt to prevent, as much as possible, the occurrence of postoperative neurological deficits resulting from unintended injury to white matter tracts both motor and sensitive as well as sensory (3, 4).

Anatomical studies, utilizing Klingler's method of brain fixation (5–9) have demonstrated that the lateral wall and the roof of the lateral ventricle are covered by optic radiations projecting from the lateral geniculate nucleus of the thalamus to the calcarine fissure. In contrast, its medial wall, in the area of the ventricular trigone, is free of them, a knowledge that has influenced the recommended surgical approach for resection of intraventricular tumors located in the lateral ventricle (4).

Homonymous hemianopia is one of the potential visual deficits that can be incurred by surgical approaches to the lateral ventricle made through its lateral or superior walls. In fact in 2.4% of a large series of homonymous hemianopia cases, this deficit resulted as a complication of brain surgery (10). The following case report highlights the persistence of this problem.

## Case Report

**Figure 1:**
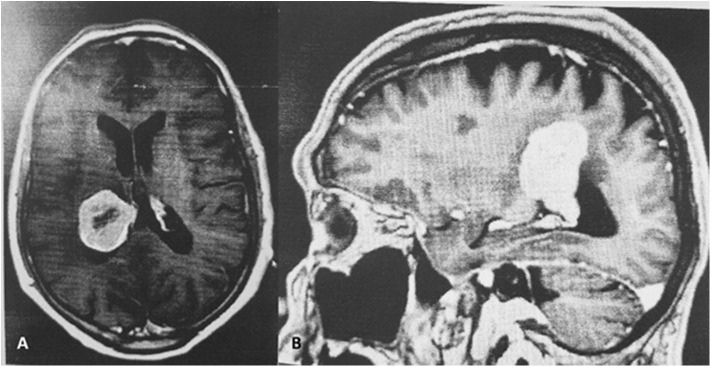
**(A,B)** Enhancing centrally necrotic intraventricular 3.8 × 3 × 4.5 cm mass in the posterior right lateral ventricle.

A. D. a 78 year-old right handed Caucasian male, a retired physician, had experienced, for the 3 years prior to his hospital admission, recurrent episodes of transient visual symptoms. These consisted exclusively in the occurrence of colorless mesh-like flickering lights in his right homonymous visual fields. These photopias were not induced by any particular stimulus, showed no movement, but their light intensity pulsated in a rhythmic pattern. These were not associated with auditory phenomena, nor were they preceded, associated with or followed by headache. These episodes lasted for 1–2 min and cleared spontaneously only to recur after variable periods of time. Because these episodes were not associated with any change in his visual acuity nor with the perception of a visual field defect A. D. did not seek, during that period of time, an ophthalmological evaluation. Only when the patient's wife reported some personality changes consisting in sleepiness and unusual sudden mood changes together with a mild gait imbalance, a cerebral MRI with Gadolinium was done and demonstrated an intraventricular meningioma in the right lateral ventricle, associated with edema in the right parietal lobe (Figures 1A,B).

The patient was admitted to a neurosurgical referral center. There his general physical and neurological examinations, including visual field testing by confrontation, detected no abnormalities. A chest x-ray, EKG, complete blood count, electrolytes, BUN, creatinine, liver function tests, and coagulation profile were all within normal limits. He was then brought to the operating room where a right posterior parietal craniotomy was performed in the prone position. A transcortical approach to the right lateral ventricle with image guidance assistance was carried out and a Vycor tubular retractor was positioned into that tract. Under the operating microscope the tumor was visualized and a biopsy was taken that revealed a meningioma grade 2. The tumor was debulked with the aid of an ultrasonic aspirator and it was eventually completely removed. A few veins attached to the tumor capsule were coagulated but no significant bleeding occurred throughout the procedure.

**Figure 2:**
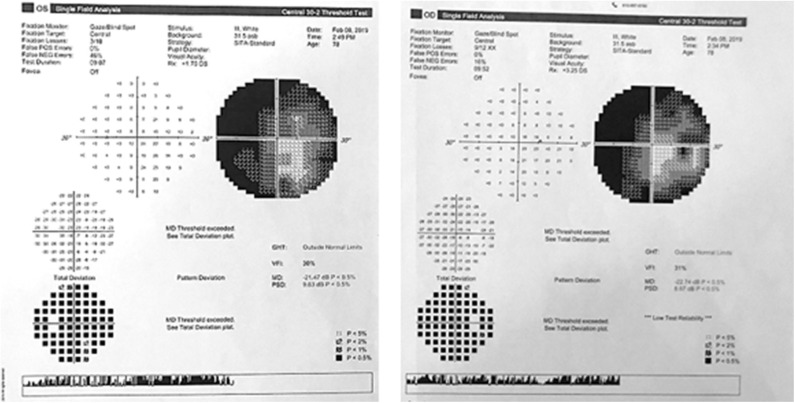
Goldman visual field test showing an incomplete homonymous hemianopia.

Post-operatively the patient reported a left homonymous hemianopia, a marked worsening in a preoperatively existing mild bilateral hypoacusia, together with some gait imbalance. His follow-up course showed a gradual improvement in his gait and the hypoacusia eventually returned to its preoperative baseline, but the homonymous hemianopia persisted. A Goldman visual field test confirmed an incomplete left homonymous hemianopia (Figure 2).

For the first 2 months after the surgery the homonymous hemianopia manifested itself simply by the lack of vision in the affected areas of the visual hemi fields. Thereafter, AD reported that some visual activity developed in the form of visual hallucinations that presented spontaneously, with no obvious triggering stimulus and were not associated with any change in his mental status. According to the patient's description, these visual hallucinations presented only within the hemianopic visual fields, lasted for a short period of time and disappeared spontaneously, only to recur after variable periods of time. These were experienced by the patient as clearly unreal and caused him no anxiety nor other emotional effects. Consequently, these could be considered to be pseudo hallucinations. According to the patient these presented in three different formats which seemed to follow each other in a certain sequence. Initially they consisted of the vision of geysers of vapor, constantly growing or shrinking, and changing their position and direction. These were monochromatic in a light gray tone and lasted for a few seconds at a time, only to recur after minutes or hours. This format persisted for 5 days after which it eventually disappeared, not to reappear again. It was followed by a second type of visual activity which consisted of the vision of a small colorless bright dot of light, a phosphene, which appeared at any point within the affected visual fields. During each episode, the visual dot did not change in size or location, it would last for a few seconds before it disappeared. It could reappear after variable periods of time in a different location but with identical characteristics. Its occurrence gradually decreased in frequency and it was eventually replaced by a black dot. This one did not seem to change in size or position between episodes and rather than a pseudo hallucination it appeared to represent a sequelae of the damaged visual pathway. It has persisted unchanged up to the time of this writing.

Finally a third type of visual phenomena started to occur about a month after the ones already described. It consisted in the simultaneous visual replication, within the hemianopic visual fields, of the image been visualized in the unaffected visual fields. This momentary photocopy [Allaesthesia (11)] would rapidly disappear. This transient visual phenomena was polychromatic, its colors matching exactly the ones present in the scene been seen by the intact visual fields. This type of visual phenomena could recur at various times during the day and persisted for about a month, after which eventually subsided and has not recurred since. Neither one of these three types of visual phenomena was associated with any simultaneous auditory phenomena. The timing of their presentation and their apparent sequence would suggest that these phenomena might have been the effect of the ongoing cortical and subcortical healing process in the affected areas. Furthermore, the fact that all these visual phenomena, including the preoperative photopias, completely subsided within a relative short time after the surgery, suggests an epileptic basis for them.

A CT scan with contrast infusion done 4 months after the surgery, confirmed the complete resection of the meningioma.

## Discussion

Homonymous hemianopia can be the result of diverse pathological conditions affecting the retrochiasmal visual pathway at any point along its course. This visual field defect is a significant one because it has financial and legal implications since it precludes driving and impairs reading and the use of a computer, thus affecting the quality of life and the capacity to work.

In the case reported herewith, the absence of a relative afferent pupillary defect essentially rules out the optic tract as the site of the causative lesion while the optic radiation, and in particular its posterior bundle, that runs along the roof of the lateral ventricle (7–9), would appear to be the most likely site for that injury. This could have been the result of either actual transection of part of the optic radiation during the surgical approach itself or otherwise by trans-ependymal optic radiation trauma during tumor removal (12). Nevertheless, and in spite of these not been absolute criteria (13), the absence of other associated neurological deficits (14) the development of formed complex hallucinations (15), the congruency of the visual field defect (13, 14) and its peripheral location (16) would argue instead for the anterior striate cortex, at the juncture of the parieto-occipital and calcarine fissures (Brodman areas 18 and 19), as the anatomical site responsible for this complication, an area that was in jeopardy during the surgical approach undertaken in this case. Furthermore, the lack of any intrinsic well-defined color as an integral part of these visual phenomena also support Brodman areas 18 and 19 as the causative site of that injury, since the areas involved in colored vision are located away in Brodman areas 37 and 39 (17). This visual deficit could have been avoided by utilizing a transcallosal avenue instead, thus obviating injury to Brodman areas 18 and 19 and making unnecessary the use of the Vycor retractor because the exposure would have been wider.

Because the visual pathway has significant variability in its anatomical position (11) and its fibers are not visible under the operating microscope, the development of diffusion-weighted single-shot STEAM MRI sequence, has permitted the “*in vivo*” mapping of the visual pathway (11, 18) thus allowing the preoperative determination of its exact location and a better selection of the most appropriate operative approach. Moreover, Diffusion tensor MRI has also been recently integrated into neuro-navigational systems (3) enabling the intraoperative visualization of these fiber tracts. Both of these advances should significantly help in decreasing the occurrence of unintended post-operative visual deficits.

## Conclusion

This report highlights the need to consider visual hallucinations as an important symptom that requires a thorough investigation. It also stresses the fact that although the use of advanced and sophisticated surgical tools is important, the selection of the most appropriate surgical approach in any given case remains crucial.

## Ethics Statement

Written informed consent was obtained from the individual(s) for the publication of any potentially identifiable images or data included in this article.

## Author Contributions

AD contributed with the original conception of the case report, reviewed the related literature, and wrote the first draft. MC evaluated the patient, gathered the diagnostic material, reviewed the first draft, and made some changes in it. Both authors read the final version of the work, approved its submission, and are accountable for all aspects of the work.

## Conflict of Interest

The authors declare that the research was conducted in the absence of any commercial or financial relationships that could be construed as a potential conflict of interest.
